# Unravelling the Digestibility and Structure–Function Relationship of Lentil Protein Through Germination and Molecular Weight Fractionation

**DOI:** 10.3390/foods14020272

**Published:** 2025-01-16

**Authors:** Armin Mirzapour-Kouhdasht, Samaneh Shaghaghian, Marjan Majdinasab, Jen-Yi Huang, Marco Garcia-Vaquero

**Affiliations:** 1 Section of Food and Nutrition, School of Agriculture and Food Science, University College Dublin, Belfield, Dublin 4, Ireland; amirzapo@purdue.edu; 2 Department of Food Science, Purdue University, West Lafayette, IN 47907, USA; huang874@purdue.edu; 3 Department of Food Science and Technology, School of Agriculture, Shiraz University, Shiraz 71441-65186, Iran; samaneh.shaghaghian.1@ulaval.ca (S.S.); majdinasab@shirazu.ac.ir (M.M.); 4 Department of Food Science, University of Laval, Quebec, QC G1V0A6, Canada

**Keywords:** lentil seeds, protein digestibility, ACE-I inhibitory activity, antioxidant, germination

## Abstract

This study explores for the first time the impact of a 6-day germination process on the structure (FTIR), antioxidant activity, nutritional/safety attributes (ACE-I inhibitory activity, digestibility, and cytotoxicity), and functional properties of fractions of variable molecular weight (W > 5 kDa; 3 kDa < MW < 5 kDa; and MW < 3 kDa) isolated from proteins extracted from lentils. FTIR results indicated a substantial increase in β-sheet contents during germination. The digestibility of proteins increased from day 0 (16.32–17.04%) to day 6 of germination (24.92–26.05%) with variable levels of digestibility depending on their MW. ACE-I inhibitory activity improved during germination in all fractions, reaching IC_50_ values of 0.95, 0.83, and 0.69 mg/mL after 6 days of germination. All antioxidant activities analyzed notably increased, particularly in low-MW fractions (MW < 3 kDa). The functional properties of low-MW fractions were also the most promising, displaying the highest water and fat binding capacities and emulsifying and foaming capacities but lower foaming and emulsifying stability compared to high-MW fractions. Cytotoxicity tests on L929 cells revealed the slight adverse effects of low-MW fractions during germination. This study provides insights into the enhanced nutritional and functional attributes of lentil proteins following germination, emphasizing their potential application in functional foods.

## 1. Introduction

Lentils (*Lens culinaris*) are a pulse crop belonging to the *Leguminosae* family and are one of the most highly consumed pulses around the globe [1]. The consumption of lentils has been linked to multiple nutritional benefits, including cholesterol- and lipid-lowering effects, and reducing the risk of cancer and type-2 diabetes, facts that could be attributed to their high dietary fiber and phenolic contents and to the high antioxidant capacity of the compounds produced by this crop [2].

The relatively high protein content of lentils, ranging from 24 to 30% (*w*/*w*), has recently attracted the attention of the food industry aiming to use lentil protein flours to fortify with plant protein novel food formulations [1,3]. Previous studies have demonstrated that among different pulses, lentils also contain high-quality protein and that its consumption is linked to antihypertensive effects [4]. Lentils are considered a rich source of isoleucine, leucine, lysine, threonine, phenylalanine, and valine compared to other crops while being deficient in methionine and cysteine [5]. The large amount of phenylalanine in lentils has been linked to an increased production of tyrosine which is needed for the formation of epinephrine and norepinephrine, contributing to improving neurotransmission activity under stress conditions [5,6]. Whereas a healthy adult synthesizes tyrosine from phenylalanine, a young child has not yet developed the enzyme phenylalanine hydroxylase needed for this conversion. Thus, for this sector of the population, tyrosine is an essential amino acid. This phenomenon can also occur in various pathological conditions [7]. In addition, branched-chain amino acids (BCAAs: leucine, valine, and isoleucine) are particularly important in older adults. BCAAs have a physiological role during protein synthesis, metabolism, food intake, and aging. Many studies have contradictory conclusions concerning the relationship between the blood levels of BCAAs or the dietary manipulation of these amino acids and changes appreciated during aging in body composition, sarcopenia, obesity, insulin, and glucose metabolism [8].

Lentil proteins are generally extracted by conventional methods that include a two-stage process, alkaline extraction (pH 8–11) followed by isoelectric precipitation (pH 4–5) [6]. Under these conditions, approximately 70% (*w*/*w*) of lentil protein is composed of globulins or salt-soluble protein [9] that can be further classified as vicilin (7S), with a molecular weight (MW) of 40–70 kDa and legumin (11S) with a MW of 320–380 kDa. The ratio of 7S/11S can change depending on the lentil variety under study; however, it is regularly described as being around 2.78 [1,9].

Despite all the aforementioned benefits of lentil seeds and their derived protein isolates, lentils, similarly to other pulses, may contain some anti-nutritional compounds (ANCs), such as tannins, lectins, phytic acid, and protease inhibitors, which may reduce their nutritional benefits. However, the process of protein extraction (alkali followed by acid) has been reported to alleviate the adverse effects of ANCs, improving the nutritional value and digestibility of lentil proteins [9]. Moreover, germination is a traditional process and a re-emerging trend in the food industry to unlock the nutritional potential of seeds by making them more readily available for human consumption [10,11]. Thereby, S. Santos, Silva, MP Valente, Gruber, and W. Vasconcelos [4] reported a significant increase, of around 30%, in protein content in different lentil varieties after sprouting.

Multiple enzymes are activated during the process of the germination of seeds, contributing to the transformation of complex molecules (carbohydrates, proteins, and fats) into small forms that are easier to digest and absorb by the human body; thus, variations in the molecular weight (MW) and properties of the protein ingredients achieved from these seeds are likely to change during the process of germination. Germination has been previously reported to increase the bioavailability of nutrients, such as vitamins, minerals, amino acids, and antioxidants, present in seeds [10,11]. From a food technology perspective, germination can be used to enhance the functional properties of the ingredients derived from these seeds. Germinated seeds can be used in bread-making to increase the protein quality and improve the texture of the final baked products. The seeds can also be sprouted and used as ingredients in plant-based milk alternatives or energy bars [10,11]. Aligned with this concept, the germination of lentils has been described as having positive changes in nutrient availability and the digestibility of the seeds, reducing anti-nutrients, and increasing bioactive compounds and the nutritional value of their proteins [12,13], making them an interesting ingredient for the development of functional foods.

This study investigated the nutritional, physicochemical, and functional changes in lentil proteins extracted from seeds during a 6-day germination period. The proximate composition of lentils (moisture, protein, lipid, carbohydrate, and fiber contents) and changes in their structure—measured by Fourier transform infrared (FTIR) analysis—were determined during the 6-day germination period. Moreover, fractions of variable MW were generated from all the protein isolates during all stages of germination to examine their digestibility, antioxidant activity (DPPH radical scavenging, ferrous chelating activity, hydroxyl radical scavenging activity, and ferric reducing antioxidant power), cytotoxicity (L929 cells), and functional properties (water/oil binding capacity and emulsifying and foaming properties) to provide a complete perspective of the changes induced by lentil proteins during the process of germination that could have an impact for the development of value-added products from different seed sprouts in the food industry.

## 2. Materials and Methods

### 2.1. Chemical Reagents

Pepsin from porcine stomach mucosa pepsin (3200 U/mg) (SRE0001) and pancreatin from porcine pancreas (P1750) were purchased from Merck (Arklow, County Wicklow, Ireland). The angiotensin-converting enzyme-I (ACE-I) inhibition assay kit-WST was purchased from Dojindo Laboratories (Kumamoto, Japan). L929 cells (mouse fibroblast, subcutaneous connective tissue) were obtained from the European Collection of Animal Cell Cultures (Salisbury, UK). All other chemicals and reagents were of analytical grade.

### 2.2. Seeds and Germination Process

**Figure 1:**
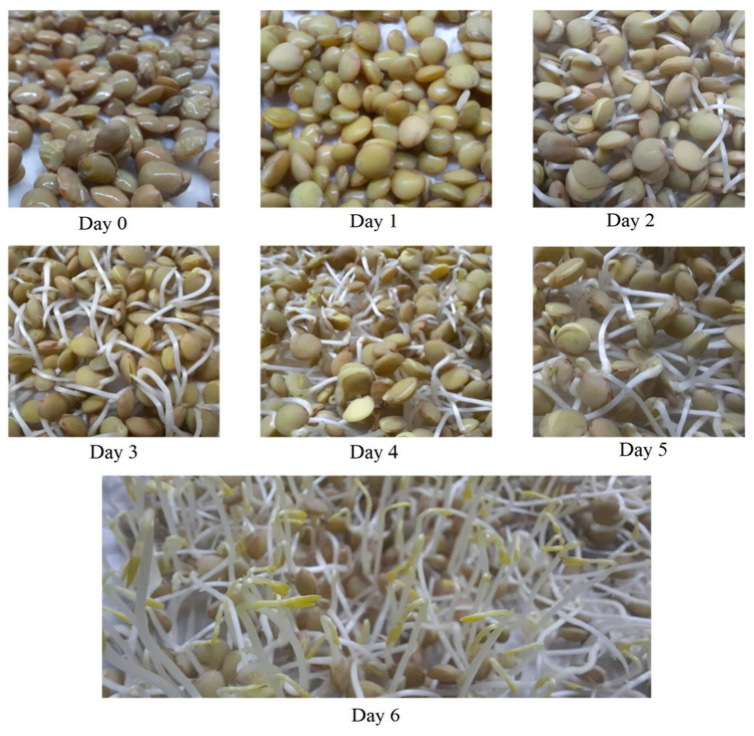
The evolution of lentil seeds during the germination process over a 6-day period.

**Figure 2:**
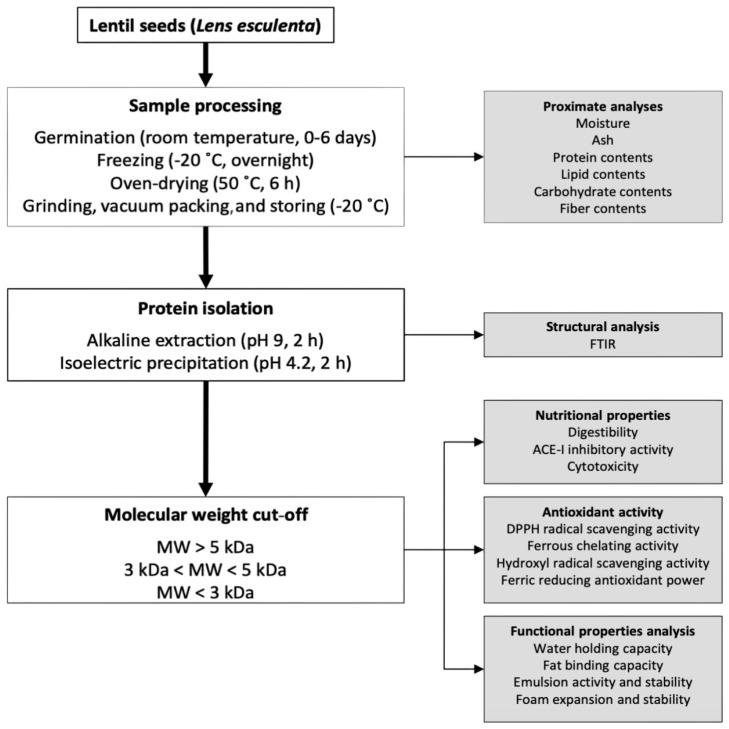
A schematic flow diagram illustrating the experimental design followed during this study.

Lentil seeds (*Lens esculenta*) were purchased from a local market in Shiraz, Iran. All seeds were washed with distilled water to remove impurities. The germination process was performed following the procedure described by Fouad and Rehab (2015) [14] with slight modifications. The lentil seeds were soaked in distilled water at a ratio of 1:10 (*w*/*v*) overnight. The water from this soaking was then discarded, and the seeds were wrapped using a wet cotton cloth and kept in dark conditions. The seeds were irrigated daily with fresh distilled water for 6 days and the cotyledon emergence dynamics and changes during that period can be appreciated in Figure 1. Samples were selected at 1-day intervals and frozen at −20 °C to cease germination. After thawing, the samples were oven-dried (50 °C, 6 h) and grinded (Kyocera, CM-20C SF, Kyoto, Japan). All samples were vacuum packed and stored at −20 °C for further analyses according to the experimental plan detailed in Figure 2.

### 2.3. Proximate Analyses

Moisture and ash contents were determined using the official method of analysis, AOAC.942.05, as described by Horowitz (2000) [15]. The protein content of the samples was assessed by the Kjeldahl method (BUCHI Labortechnik AG, Flawil, Switzerland) using a nitrogen-to-protein conversion coefficient of 6.25 [16]. Lipid contents were measured using Soxhlet apparatus, following the procedure outlined by Connolly, Piggott, and FitzGerald (2013). Briefly, 1 g of the samples was mixed with 100 mL of hexane (68 °C, 6 h) and the solvent evaporated in a rotary evaporator (Stuart, RE401, UK). The carbohydrate content of the samples was determined following the method as described by Masuko et al. (2005) [17]. Briefly, 30 µL of the samples or a standard (glucose, 10–50 mg/mL) was mixed with 150 µL of concentrated sulfuric acid (98.5%) and 30 µL of 5% phenol solution and shaken for 2 min. The mixtures were incubated in a water bath (90 °C, 5 min), cooled down at room temperature for 10 min, and agitated for 10 min before the absorbance of the reactions was read at 490 nm using a spectrophotometer (BioTek, Winooski, VT, USA).

The fiber content of the samples was evaluated as outlined by Lee, Prosky, and Vries (1992) [18]. Briefly, 1 g of the samples was mixed with 40 mL of maleate buffer (50 Mm, pH 6.0) containing 2 mM CaCl_2_, 50 U/mL porcine pancreatic α-amylase, and 3.4 U/mL amyloglucosidase, and the solutions were incubated (37 °C, 16 h). The pH of the mixtures was adjusted to 8.2 before adding 0.1 mL of *Bacillus licheniformis*-derived protease (350 U/mL), and the samples were incubated (60 °C, 30 min), cooled down to room temperature, and mixed with 4 volumes of 95% ethanol, and the filtrates were oven-dried (105 °C, overnight). The fiber contents were calculated using Equation (1).

(1) $$Fiber\;content\;\left(\%\right)=\frac{W1\;-W2\;}{W1}\times \;100$$

W_1_ and W_2_ are the initial weight of the sample and weight of the dried filtrate, respectively.

### 2.4. Protein Isolation Process

Proteins were isolated from the powdered samples as described by Suliman, El Tinay, Elkhalifa, Babiker, and Elkhalil (2006) [19] with slight modifications. Briefly, 30 g of the samples was suspended in 500 mL of distilled water, and their pH was adjusted to 9 using 1 M NaOH. After mixing for 2 h, the samples were centrifuged (3000× *g*, 30 min). The supernatants were collected and the pH was adjusted to 4.2 using 1 M HCl. The solutions were mixed for 2 h before centrifugation (10,000× *g*, 20 min); the precipitated proteins were rehydrated, neutralized to a pH of 7, freeze-dried, and stored at −20 °C for further experiments.

### 2.5. Molecular Weight Cut-Off Fractionation of Protein Isolates

Protein isolates were re-dissolved in deionized water (1:10 *w*/*v*) and sequentially filtered through 3 and 5 kDa centrifugal ultrafilter tubes (Millipore, Sigma-Aldrich, St. Louis, MO, USA). The process resulted in 3 different MWCO fractions of variable MW: (1) MW > 5 kDa; (2) 3 kDa < MW < 5 kDa; and (3) MW < 3 kDa. All the fractions were freeze-dried and stored at −20 °C for further experiments.

### 2.6. Fourier Transform Infrared (FTIR) Analysis

The secondary structural compositions of germinated and ungerminated lentil protein isolates were analyzed using a Fourier transform infrared (FTIR) spectrometer equipped with an attenuated total reflectance cell (Tensor II, Bruker, Germany). The results were recorded three times, using a wavelength range of 4000 to 400 cm^−1^ (mid-infrared region) for each sample. The second-derivative analysis was conducted using OriginPro software (version 9.0, 2013) computing the areas of different spectral components, showing the relative proportion of secondary structures.

### 2.7. Nutritional Properties

#### 2.7.1. Digestibility

The digestibility of samples was assessed following the procedure outlined by Marrion et al. (2005) [20] with slight modifications. Samples, with an approximately 40 mg nitrogen content, were digested with pepsin (1:60,000, 30 min, pH 2, and 37 °C). Subsequently, the samples were treated with pancreatin (pH 7.5, 37 °C) inside dialysis bags for 6 h. The digestibility of the samples was calculated using Equation (2).

(2) $$Digestibility\;(\%)\;=\;\frac{N\;in\;dialysates\;(mg)}{N\;in\;sample\;(40\;mg)}\times \;100$$

#### 2.7.2. Angiotensin-Converting Enzyme I (ACE-I) Inhibitory Activity

The ACE-I inhibitory activity of all samples was tested using the ACE-I inhibition kit-WST (Dojindo EU GmbH, Munich, Germany) following the manufacturers’ recommendations. Briefly, after preparing enzyme B solution (by dissolving it in 2 mL of deionized water), 1.5 mL of enzyme B solution was added to enzyme A, generating the enzyme working solution. Enzyme C and coenzyme were both dissolved in 3 mL of deionized water and 2.8 mL of each solution was combined, producing the indicator working solution. Blank 1 was established by adding 20 μL of deionized water and 20 μL of substrate buffer. Blank 2 was created by adding 40 μL of deionized water and 20 μL of substrate buffer. The inhibitors were tested by adding 20 μL of the unknown samples (0, 0.25, 0.5, 0.75, 1, 1.5, and 2 mg/mL) or positive control (captopril solution, 15 ng/mL) with 20 μL of substrate buffer. Afterwards, 20 μL of enzyme working solution was added to each inhibitor and negative control well to initiate the enzymatic reaction. The microplates were incubated (dark conditions, 37 °C, 1 h) under constant agitation (50 rpm). Following incubation, 200 μL of indicator working solution was added to each well and the plates were incubated for 10 min at room temperature. The absorbance of the samples was read at 450 nm using a spectrophotometer (BioTek, Winooski, VT, USA). The ACE-I inhibitory activity of the samples was calculated using the following equation (Equation (3)).

(3) $$ACE-I\;inhibition\;\left(\%\right)=\frac{\left(AB1\;-\;AI\right)}{AB1\;-\;AB2\;}\;\times 100\;$$

where A_B1_, A_I_, and A_B2_ are the absorbance of the control, sample, and blank, respectively.

The half-maximal inhibitory concentration (IC_50_) was considered the concentration of the sample at which 50% of ACE-I activity was inhibited.

#### 2.7.3. Cytotoxicity

Cytotoxicity was determined using L929 (mouse fibroblast, subcutaneous connective tissue) following the method of Benjakul, Karnjanapratum, and Visessanguan [21] with slight modifications. Briefly, normal L929 cell lines were cultivated in DMEM and supplemented with 10% fetal bovine serum. The cells were then placed in a 5% CO_2_ incubator at 37 °C, and cell line cultures with a density of 2 × 10^4^ cells/mL were placed in microplate wells (100 µL per well). Subsequently, 100 µL of each sample at a concentration of 1 mg/mL was introduced into the wells and incubated (37 °C, 72 h). The cell viability assessment was performed using the MTT cell proliferation kit I (Roche Diagnostics; Burgess Hill, West Sussex, UK) and following the manufacturer’s recommendations. Briefly, after the incubation period, 10 μL of the MTT labeling reagent (final concentration 0.5 mg/mL) were added to each well and incubated (4 h, 37 °C, 5% CO_2_). Subsequently, 100 μL of solubilization buffer were added to each well, and the plates were incubated overnight (37 °C, 5% CO_2_). The solubilization of the purple formazan crystals was measured by reading the absorbance of the wells at 570 nm with a spectrophotometer (BioTek, Winooski, VT, USA).

### 2.8. Antioxidant Activity

#### 2.8.1. DPPH Radical Scavenging Activity

DPPH radical scavenging activity was evaluated using the method described by Ambigaipalan and Shahidi (2015) [22] with slight modifications. Briefly, 200 μL samples (1 mg/mL) were mixed with 800 μL of 0.1 mM DPPH solution in 95% methanol. The mixtures were incubated in dark conditions (30 min) and the absorbance of the reactions was read at 517 nm using a spectrophotometer (BioTek, Winooski, VT, USA). Distilled water and ascorbic acid were used as negative and positive controls, respectively.

The DPPH radical scavenging activity (%) of the samples was calculated using Equation (4).

(4) $$DPPH\;radical\;scavenging\;\left(\%\right)=\frac{AC\;-\;AS}{AC}\times \;100$$

where A_C_ and A_S_ are the absorbance of the control and samples, respectively.

#### 2.8.2. Ferrous Chelating (FC) Activity

FC activity was determined as detailed by T. Wang, Jonsdottir, and Ólafsdóttir (2009) [23]. Briefly, 1.74 mL of deionized water was added to 200 µL samples (1 mg/mL), the positive control (Na_3_EDTA solution at 1 mg/mL), or blanks (water) and mixed with 20 µL of 2 mM FeCl_2_ and 40 µL of 5 mM ferrozine solutions. The mixtures were shaken thoroughly for 10 min at room temperature. The absorbance of the reactions was measured at 562 nm using a spectrophotometer (BioTek, Winooski, VT, USA). Na_3_EDTA served as the reference standard, and 100 μL of distilled water was employed instead of the samples as a control. Instead of the ferrozine solution, 10 μL of distilled water was utilized as a blank. The FC activity (%) of the samples was calculated using the following equation (Equation (5)).

FC (%) = [A_C_ − (A_S_ − A_B_)] × 100 (5)

where A_C_ refers to the absorbance of the control, A_S_ to the absorbance of the sample or standard, and A_B_ to the absorbance of the blank.

#### 2.8.3. Hydroxyl Radical Scavenging Activity

Hydroxyl radical scavenging activity was determined following the method as described by B. Wang et al. (2013) [24]. Briefly, 2 mL samples (1 mg/mL) were mixed with 1.865 mM of a 1,10-phenanthroline solution at a ratio of 1:2 (*v*/*v*). Thereafter, 750 µL of FeSO_4_·7H_2_O solution and 1 mL of H_2_O_2_ (0.03% *v*/*v*) were added to the mixtures and incubated (37 °C, 1 h). The absorbance of the samples was recorded at 536 nm using a spectrophotometer (BioTek, Winooski, VT, USA). The negative control (n) and blank (b) were established using the reaction mixture without the inclusion of samples (s) and H_2_O_2_, respectively. The hydroxyl radical scavenging activity (%) of the samples was calculated using the following equation (Equation (6)).

(6) $$Hydroxyl\;radical\;scavenging\;\left(\%\right)=\frac{As\;-\;An}{Ab\;-\;An}\times \;100$$

where A_s_, A_n_, and A_b_ refer to the absorbance of the sample, negative control, and blank, respectively.

#### 2.8.4. Ferric Reducing Antioxidant Power (FRAP)

FRAP was determined as described by Oyaizu (1986) [25] with the modifications performed by Karawita et al. (2005) [26]. Briefly, 2.5 mL of each sample (1 mg/mL) or trolox (500 µM), as a positive control, was mixed with equal volumes of 200 mM phosphate buffer (pH 6.6) and 1% potassium ferricyanide. The mixtures were incubated (50 °C, 20 min) and allowed to cool down to room temperature; the addition of 2.5 mL of trichloroacetic acid (10%) followed. The mixtures were then centrifuged (4200× *g*, 10 min). An aliquot (5 mL) of the resultant supernatant was spiked with an equal volume of deionized water and 1 mL of ferric chloride solution (0.1%, *w*/*v*). The absorbance of the reactions was measured at 690 nm using a spectrophotometer (BioTek, Winooski, VT, USA).

### 2.9. Functional Properties

#### 2.9.1. Water Holding and Fat Binding Capacities

The water holding capacity (WHC) and fat-binding capacity (FBC) were measured as described in Mirzapour-Kouhdasht, Moosavi-Nasab, Kim, and Eun (2021) [27]. For the WHC, 100 mg samples were combined with 10 mL of distilled water and the mixtures were gently agitated (100 rpm, 1 h, room temperature). Subsequently, these mixtures were centrifuged (2000× *g*, 25 min), the supernatants were drained, and the WHC was quantified by comparing the initial weight of the tubes to their weight after draining. In the case of the FBC, 100 mg samples were combined with 2 mL of olive oil and the mixtures were allowed to stand for 30 min at room temperature with intermittent shaking every 10 min. Subsequently, the mixtures were centrifuged (2000× *g*, 25 min) to drain the oil and the FBC was determined by comparing the initial weight of the tubes to their weight after draining.

#### 2.9.2. Emulsion Activity and Stability

The emulsion activity index (EAI) and emulsion stability index (ESI) were assessed as reported by Mirzapour-Kouhdasht et al. (2021) [27]. A total of 30 mL of each sample (0.1% *w*/*v*) was blended with 10 mL of soybean oil using a homogenizer (CAT Unidrive 1000, CAT Scientific, Paso Robles CA, USA). After homogenization at 0 and 10 min, 100 µL of the emulsion was diluted 100-fold using SDS solution (0.1% *w*/*v*). The absorbance of the samples was then measured at 500 nm using a microplate reader. The EAI (m^2^/g) and ESI (min) were calculated using Equation (7) and Equation (8), respectively.

(7) $$EAI\;(m^{2}/g)=\frac{2\;\times \;2.303\;\times \;A}{0.25\;\times \;Cp}$$

(8) $$ESI\;(min)=\frac{A0\;\times \;\Delta t}{\Delta A}$$

where A denotes the absorbance of the mixtures at 500 nm; Cp denotes the protein concentration, A_0_ refers to the initial absorbance, and A_10_ refers to the absorbance at 10 min post-homogenization. Δt corresponds to a time interval of 10 min, while ΔA represents the disparity between A_0_ and A_10_.

#### 2.9.3. Foam Expansion and Stability

Foam expansion (FE) and foam stability (FS) were determined using the method described by Shahidi, Han, and Synowiecki (1995) [28]. Following the homogenization of each sample (2% *w*/*v*, 1 min, room temperature), the samples were transferred to cylindrical vessels. Alterations in the sample volume were gauged after 30 and 60 min. FE (%) and FS (%) were calculated using Equation (9) and Equation (10), respectively.

FE (%) = (V_A_ − V_B_)/(V_B_) × 100 (9)

FS (%) = (V_30/60_ − V_B_)/(V_B_) × 100 (10)

where V_A_ is the total volume after homogenizing, V_B_ is the total volume before homogenizing, and V_30/60_ is the total volume after 30 and 60 min.

### 2.10. Statistical Analyses

All analyses were performed in duplicate, and cytotoxicity was assayed in triplicate. The normality of the data sets was confirmed by Kolmogorov–Smirnov tests (*p* > 0.05) and the equal variances were tested using Levene’s tests (*p* < 0.05) indicating that the null hypothesis of equal variances could not be accepted. One-way multivariate analysis of variance (one-way MANOVA) was used to check differences in the proximate composition of the seeds based on the germination period. Two-way multivariate analysis of variance (MANOVA) was used to analyze the influence of germination, MW purification, and interaction between both factors in digestibility, ACE-I inhibitory activity, cytotoxicity, antioxidant activities, and functional properties. Games–Howell post hoc tests were used in all cases to analyze in detail the differences within the groups. All data were analyzed using SPSS version 23 (IBM, North Castle, NY, USA). In all cases, the criterion for statistical significance was *p* < 0.05.

## 3. Results and Discussions

### 3.1. Proximate Analyses

The proximate composition (moisture, protein, fat, carbohydrate, fiber, and ash contents) of lentils from days 0 to 6 during their germination is summarized in Table 1. There were statistical differences in all parameters during germination (*p* < 0.001; F = 2.990). Overall, the fat and carbohydrate contents in the lentils decreased significantly over the germination period, reaching minimum levels of ≈ 1% of fat and 37% of carbohydrates in days 5–6. An opposite behavior was appreciated in the case of the moisture, protein, fiber, and ash contents, with a tendency to increase during the process of germination, reaching maximum levels of approximately 12–13% moisture, 25% protein, 21% fiber, and 3–4% ash contents around days 4–5 of germination.

These results are similar to those of Fouad and Rehab [14], who reported an increase in moisture, protein, ash, and fiber and a decline in carbohydrate and fat content in lentils during their germination. Moreover, Ghumman, Kaur, and Singh [29] reported an increase in protein contents in lentils during 4 days of germination. However, contrary to the findings of this study, the authors reported a decrease in the levels of ash during germination. The changes appreciated in the protein of seeds during germination could be related to the production of enzymes needed during the process of germination, leading to increased amino acid production and the degradation of high-MW proteins into small peptides [29].

Lentils, like other pulses of this family, may contain variable amounts of ANCs, including tannins, lectins, phytic acid, and protease inhibitors that can potentially reduce their nutritional benefits. However, the protein extraction processes used in this study (alkaline extraction and isoelectric precipitation) have been previously reported as being effective strategies to alleviate the adverse effects of ANCs and to improve the nutritional value and digestibility of these proteins. This could possibly be attributed to the denaturation or damage of ANCs at high pH values [9,30]. Moreover, the final neutral pH of the lentil protein isolates provides a net negative charge on the proteins, which can lead to weak protein–phytic acid binding and the leaching out of phytic acid during the protein extraction process [31].

### 3.2. Structural Characteristics of Protein Isolates

**Figure 3:**
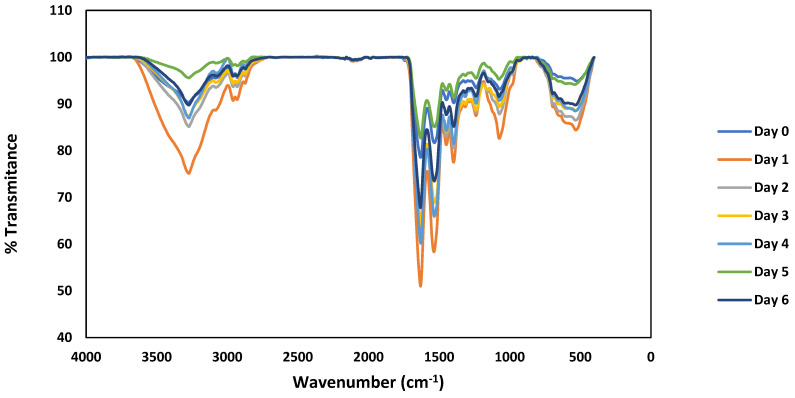
FTIR spectra of protein isolates generated from lentil seeds during germination (days 0–6). Results were recorded three times for each sample.

The structural conformation of proteins affect the functionality and digestibility of these compounds [32]. Thus, changes in the secondary structural composition of proteins can alter their technological properties [33] which are critical for their future uses in the food industry. The changes in the chemical bonds and structural properties of protein isolates during the process of lentil germination were analyzed by FTIR spectroscopy (Figure 3).

Previous studies analyzing the FTIR spectra of germinated pulses have reported enhancement in the water-solubility of proteins for enzymatic activity to develop new tissues during germination [34]. The vibration of peptide bonds at the wavelength of 1700–1500 cm^−1^ indicates the protein content of lentil seeds [34]. The amide I (C=O, O-H) region (1730–1580 cm^−1^) and the amide II (N-H, C-N) region (1580–1470 cm^−1^) were analyzed to determine the relative proportions of different secondary structures by computing areas of the spectrums at ≈1630 cm^−1^ (β-sheet) as the main constituents of the protein’s secondary structure (see Table 2). Previous studies have reported that changes in the amide I region depict changes in the secondary structure of protein molecules [35].

The results of this study indicate a reduction in the β-sheet configuration of lentil proteins during germination, which is consistent with previous observations on the structural modifications of proteins during the germination of pulses. For example, the reduction in β-sheet content might correspond with increased protein solubility and digestibility, enhancing the bioavailability of nutrients. These structural changes can improve the functional properties of proteins, including changes in emulsification, foaming, and water-holding capacity, which can be of benefit for various food applications. However, it may also lead to reduced thermal stability and the potential loss of some functional properties that rely on the β-sheet structure, like gel formation [34,35]. Thus, the functional properties of these proteins were also analyzed, aiming to provide a complete range of future applications for these proteins in food formulation depending on the germination stage of the seeds.

Table 2 shows the changes in the content of β-sheets in lentil proteins during 6 days of germination. After an initial increase, there was a notable reduction in β-sheets that stabilized around days 4–6. This trend suggests a dynamic restructuring process where the initial breakdown of complex structures facilitates new tissue development and enzymatic activities, ultimately leading to a more flexible and functional protein configuration suitable for diverse food industry applications. In summary, the observed reduction in β-sheet configuration during lentil germination aligns with enhanced functional properties, making germinated lentil proteins potentially more versatile for food industry applications, while balancing improvements in digestibility and nutrient bioavailability.

### 3.3. Nutritional Properties

#### 3.3.1. Digestibility

Aiming to elucidate the impact of germination on protein digestibility, the lentil protein isolates were purified into different MW fractions, and the digestibility of different MW fractions achieved from protein isolates from lentils during the process of germination (days 0–6) are compiled in Table 3. The digestibility was affected by germination (*p* < 0.001; F = 73683.102), MW purification (*p* < 0.001; F = 3709.894), and the interaction between both factors (germination*MW; *p* < 0.001; F = 67.020).

The protein digestibility after the dialysis of three MW fractions of lentil protein isolates ranged from 16.32 to 17.04%. As observed in Table 3, the digestibility of all tested fractions was increased by pepsin and pancreatin hydrolysis during sprouting. The digestibility of the fractions with MW > 5 kDa and 3 kDa < MW < 5 kDa and MW < 3 kDa of lentil seeds increased significantly (*p* < 0.05) from levels of 16.32, 17, and 17.04% to reach levels of 24.92, 25.64, and 26.05% after 6 days of germination, respectively. This indicates an increased susceptibility of the peptide bonds to digestive enzymes in the proteins as the germination of the lentil seeds progresses.

As previously mentioned, the interaction between MW and germination time was significant, as the rate of increase in digestibility over time varied across different MW fractions. The low-MW fractions had a more pronounced improvement in digestibility, indicating that both MW and germination duration significantly influence protein digestibility. The observed increase in protein digestibility during germination aligns with previous studies. Thereby, Di, Li, Chang, Gu, Duan, Liu, Liu, and Wang [35] reported similar findings and attributed these changes to enhanced protease activity, the deactivation of protease inhibitors, and protein degradation in small and soluble peptides [36]. This enhanced digestibility of lentil proteins can improve their nutritional quality and functionality for food applications, potentially making protein ingredients extracted from germinated lentils more desirable for health-conscious consumers and food manufacturers seeking ingredients with high bioavailability and improved functional properties [37].

#### 3.3.2. ACE-I Inhibitory Activity

The inhibition of ACE-I (EC 3.4.15.1) is an effective strategy for lowering blood pressure. Multiple peptides have been identified from various natural sources as inhibitors of ACE-I with potential to replace conventional drugs, such as captopril, and avoid the adverse side effects of pharmacological treatments [38].

The ACE-I inhibitory activity of different MW fractions from protein isolates from lentils during the process of germination (days 0–6) are summarized in Table 4. There were statistical differences in ACE-I inhibitory activity during germination (*p* < 0.05; F = 491.709), MW purification (*p* < 0.05; F = 1344.830), and the interaction between both factors (germination*MW; *p* < 0.05; F = 10.172). Overall, the IC_50_ of the compounds had its lowest levels in fractions of low MW within the same period of germination. Moreover, independently of the MW of the fractions analyzed, the IC_50_ of the compounds decreased with an increased germination time. The lowest activity was found in non-germinated lentil protein (IC_50_~1.49–1.03%), and the IC_50_ of the compounds decreased to reach 0.95- 0.69% after a week of germination. Thus, a low MW and germination time had a positive effect, increasing the ACE-I inhibitory activity of lentil proteins. Bamdad et al. [39] also reported that germination can release bioactive peptides with high ACE inhibitory activity. The increase in the ACE-I inhibitory activity of germinated seeds can be explained by the increased degradation of protein structures, also appreciated in the previously described FTIR results. Furthermore, previous studies have also reported an increased activity of endogenous proteases during sprouting, leading to the generation of peptides with specific MW, composition, and amino acid sequences with potential ACE-I inhibitory characteristics [40].

The IC_50_ levels of the fractions generated in this study were similar to those reported in previous research dealing with the hydrolysis of protein isolates from lentils and other pulses. In such research, protein hydrolysates generated from lentil protein hydrolysates had an IC_50_ of 0.25 mg/mL [41], red lentil protein hydrolysates had an IC_50_ of 0.44 mg/mL [42], green soybean protein hydrolysates had IC_50_ of 0.14 mg/mL [43], and lupin protein hydrolysates had an IC_50_ of 0.22 mg/mL [44].

#### 3.3.3. Cytotoxicity

**Figure 4:**
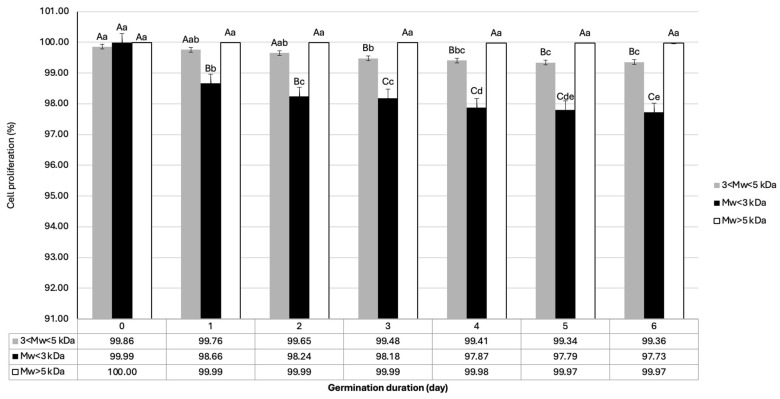
Cytotoxicity of different MW fractions achieved from protein isolates from lentils during process of germination (days 0–6) using L929 cell lines. Different uppercase letters indicate statistically significant differences (*p* < 0.05) in cytotoxicity between fractions of different MW within same germination day. Lowercase letters indicate statistically significant (*p* < 0.05) differences in cytotoxicity between different germination days at same MW. Data are shown as average of three replicates.

The effect of protein isolates of different MW fractions achieved from protein isolates from lentils in different germination stages on the proliferation of L929 cells is represented in Figure 4. The cytotoxicity of the samples was affected by the germination (*p* < 0.001; F = 192.555), MW purification (*p* < 0.001; F = 2960.312), and the interaction between both factors (germination*MW; *p* < 0.001; F = 101.532). Protein fractions of MW < 3 kDa and 3 kDa < MW < 5 kDa showed significant adverse effects. The cell proliferation remained unchanged in the presence of high-MW protein fractions (MW > 5 kDa) during the sprouting process. The lowest cell proliferation of the L929 cell line (97.63%) was related to the treatment with the MW < 3 kDa lentil protein fraction after 6 days of germination. Meaningful differences were appreciated among cell lines treated with different MW fractions achieved during germination and thus the germination process had a toxicity effect of MW < 3 kDa and 3 kDa < MW < 5 kDa fractions on normal L929 cells.

In a study by Hlosrichok and Aunpad [45], winged bean (*Psophocarpus tetragonolobus*) seeds’ protein was extracted by gamma rays and this was followed by enzymatic hydrolysis with alcalase (55 °C, 6 h). The study revealed that winged bean protein hydrolysates had no toxicity against L929 cells. These variable results could be attributed to the different protein sources used in each study. There are some types of proteins and glycoproteins in lentils called lectins that can induce cytotoxicity in L929 cell lines [46]. To the best of our knowledge, there is not enough evidence of using L929 cell lines to examine the effect of germination on the cytotoxicity of proteins derived from lentil seeds. Hence, further studies are needed to clarify the exact effects of this process and the compounds generated on cell proliferation.

### 3.4. Antioxidant Activities

**Figure 5:**
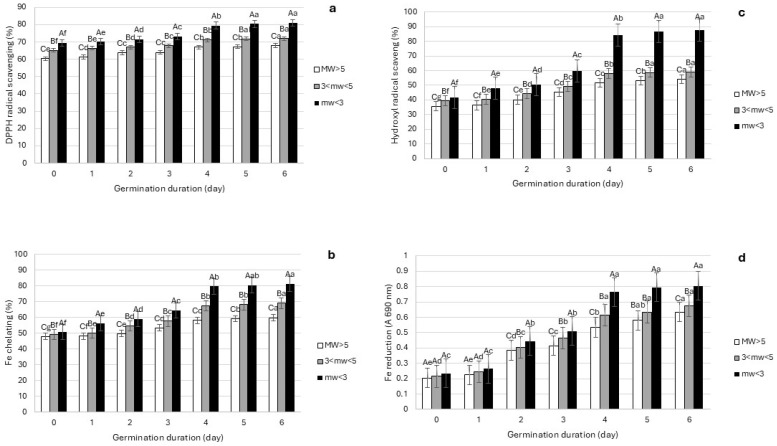
Antioxidant activities: (**a**) DPPH (%), (**b**) Fe chelating (%), (**c**) hydroxyl radical (%), and (**d**) FRAP (A) activities of different MW fractions achieved from protein isolates from lentils during process of germination (days 0–6). Different uppercase letters indicate statistically significant differences (*p* < 0.05) in antioxidant activity between fractions of different MW within same germination day. Lowercase letters indicate statistically significant (*p* < 0.05) differences in antioxidant activity between different germination days at same MW. Data are shown as average of two replicates.

The antioxidant activities of three fractions of different MW achieved from protein isolates from lentils during the first 6 days of the germination period were assessed in terms of their ability to scavenge DPPH radicals (Figure 5a), ferrous chelating activity (Figure 5b), hydroxyl radical scavenging activity (Figure 5c), and FRAP (Figure 5d). There were statistical differences in all antioxidant activities tested on the basis of germination (*p* < 0.001; F = 54.784), MW (*p* < 0.001; F = 12846.455), and the interaction between both (germination*MW; *p* < 0.001; F = 9.975).

Although low-MW fractions already had high DPPH scavenging activity before the process of germination started (<70%), this activity increased to reach over 80% after 6 days of germination (Figure 5a). This increment in antioxidant activity was not appreciated during the process of germination in the case of fractions of high MW (60% and 67% before and after germination, respectively). However, the increment was statistically significant (*p* < 0.05) when comparing the DPPH radical scavenging activity of the lower-MW fractions of the protein to non-germinated and to germinated ones. These results are aligned with those described by Fouad and Rehab [14], showing a 53% increase in radical scavenging activity after a week of the germination of lentil seeds. Moreover, when studying the antioxidant ability of different seeds during germination, previous reports have emphasized an increased level of DPPH scavenging activity during a germination period of 4–7 days in soybean, dill, and anise seeds [47]. The DPPH radical scavenging activity of various peptides achieved using protein hydrolysis have been previously reported to depend on the MW size and sequence of the peptides. Both factors are also influenced by the type of enzyme used to generate those hydrolysates [48] or the endogenous hydrolytic activities present naturally in the seeds, as well as the presence of other natural antioxidants, such as vitamin C and tocopherols [14]. Previous studies with pulses have demonstrated high levels of DPPH in protein fractions of low MW. Olagunju, Omoba, Enujiugha, Alashi, and Aluko [48] reported the highest DPPH radical scavenging activity in low-MW pancreatin–hydrolysate peptides achieved from pigeon pea protein isolates.

In the case of the ferrous chelating power of the compounds (Figure 5b), this activity can also be affected by the peptide size and amino acid composition [27]. The results of the ferrous chelating activity of lentil seeds demonstrated a marked increase in the Fe^2+^ chelating ability on day 2 of germination for all MW fractions. This increase was significantly greater in low-MW protein fractions (<3 kDa), reaching 58.97%, in comparison with the other two higher-MW fractions (54.51% in 3 < MW < 5 kDa and 49.74% in MW > 5 kDa). Balanescu, Busuioc, Botezatu, Gosav, Avramescu, Furdui, and Dinica [47] studied the reducing iron chelating power of soybean, dill, and anise seeds and their sprouts (germinated for 4 to 7 days). The authors reported that while the extracts from sprouts had a higher iron binding ability than the seeds, this activity was also influenced by the type of biomass studied. Soybean had the highest reducing iron chelating power, followed by dill and anise seeds, a fact that could be explained by their differences in their amino acid composition [47].

The hydroxyl radical scavenging activity followed a similar trend to that previously mentioned in ferrous chelating activity (Figure 5c). From day 2 of germination, all protein fractions showed a statistical increase in their capacity to scavenge hydroxyl radicals, being this increased antioxidant properties more accentuated in low-MW fractions (MW < 3 kDa). In raw lentil seeds, the hydroxyl radical scavenging was 35, 39, and 41% for fractions with MW > kDa 5 and 3 kDa < MW < 5 kDa and MW < 3 kDa; that increased to 54, 59, and 87% after 6 days of germination, respectively. The reaction between H_2_O_2_ and Fe^2+^ produces transitory and intensely reactive hydroxyl radicals with oxidative damage effects on biological macromolecules, such as DNA, proteins, and lipids [48]. Thus, antioxidants with high hydroxyl radical scavenging ability can significantly prevent oxidative stress reactions in living cells [48]. Previous studies have also reported an influence of the amino acid composition of peptides on their hydroxyl radical scavenging ability. Thereby, a higher amount of hydrophobic amino acids have been related to the higher hydroxyl radical scavenging ability of these peptides [27]. Moreover, high hydroxyl scavenging activity was reported in low-MW peptides. Thereby, the highest hydroxyl radical scavenging ability of pigeon pea protein hydrolysates was appreciated in peptides of low MW (<1 kDa) generated from ultrafiltration from full hydrolysates [48].

Measuring FRAP is another indicator of the antioxidant ability of different compounds. The presence of hydrogen or electron donor compounds (reductones) can reduce lipid peroxyl radicals and inhibit lipid oxidation [14,48]. The reported positive correlation between FRAP, hydroxyl radical scavenging, ferrous chelating activity, and the antioxidant activity of different peptide fractions was consistent with previous findings [49]. In a study conducted by Parit et al. [50], a proteomic analysis was performed on wheat (*Triticum aestivum*) seeds at 0, 8, and 16 days of germination. The study revealed that the seeds germinated for 16 days exhibited the highest FRAP value, followed by those germinated for 8 days and then the ungerminated seeds (0 days). This indicates a direct correlation between the duration of germination and the FRAP value, with longer germination periods leading to higher antioxidant capacity. As shown in Figure 5d, the FRAP of lentil protein fractions increased during the process of germination. The absorbance of low-MW fractions increased from 0.23 to 0.8 after 6 days of germination. Investigating the antioxidant activity of *Amaranthus viridis* seeds, the DPPH, FRAP, and metal chelating ability showed a remarkable rise during the process of the germination of the seeds during a period of 3 days [51]. Moreover, Fouad and Rehab [14] also reported an increase in the FRAP from 0.22 in raw lentil seeds to 0.55 in lentil seeds germinated for 6 days. In contrast to the results of this work, Olagunju, Omoba, Enujiugha, Alashi, and Aluko [48] reported a higher FRAP in high-MW peptides (>10 kDa) than in low-MW ones (1–10 kDa) in pigeon peas, attributed to differences in the amino acid composition of the different bioactive peptide fractions generated during the germination process [27].

### 3.5. Functional Properties

Lentil protein ingredients are generally described as having acceptable functional properties, allowing them to retain water and fat in food formulations and to be used as a promising emulsifier and foaming agents. However, these properties are influenced by changes in MW and surface hydrophobicity that the compounds experience during the process of protein hydrolysis [52]. A summary of the functional properties of different MW fractions achieved from protein isolates from lentils during the process of germination (days 0–6) in this study is provided in Table 5. There were statistical differences in all functional properties tested on the basis of germination (*p* < 0.001; F = 25.004), MW (*p* < 0.001; F = 7400.944), and the interaction between both (germination*MW; *p* < 0.001; F = 207.280).

#### 3.5.1. WHC and FBC

WHC and FBC are influenced by the polarity of the amino acids present in proteins/peptides [53]. WHC refers to proteins’ capacity to inhibit the liquid leakage of a product while being produced and/or stored [2]. The results of this study revealed that low-MW fractions had a higher ability to retain water, and there were significant differences between the same fractions during the process of germination until day 6, in which the low-MW fractions reached a maximum WHC level of ≈118%. Similarly, the FBC of protein isolates increased during germination and, after 6 days, the FBC of the protein isolates increased by more than 20%. Moreover, meaningful differences were observed in the ability of protein fractions with MW > 5 kDa and 3 kDa < MW < 5 kDa in holding oil, which could be due to the approximate unequal proportion of polar amino acids. Mirzapour-Kouhdasht, Moosavi-Nasab, Kim, and Eun [27] reported that the dual character of MW < 3 kDa fractions could be an asset in food systems to act as emulsifiers. Ghumman, Kaur, and Singh [29] reported similar results to those of this study. The authors outlined a significant increase in the WHC of germinated lentils’ and horsegrams’ flours that could be attributed to a rise in the content of low-MW proteins and polar groups upon germination. Other studies have also reported slight improvements in WHC in yellow pea and faba bean flours after soaking and germination (3 days) due to the exposure of hydrophilic moieties; however, few changes were observed in the case of their FBC [36].

#### 3.5.2. EAI and ESI

The EAI and ESI of proteins are important functional properties when these ingredients are needed to act as surfactants in emulsions for their use in different food formulations. The results of this study indicate that EAI values increased during germination, while the MW was inversely correlated to the EAI values. Fractions of MW < 3 kDa had the highest EAI levels of 135.42 m^2^/g after 6 days of germination, compared to any other fraction of high MW and any other germination period. However, the stability of the emulsions formed was also at its lowest (20 min). High-MW fractions were able to achieve high ESI levels, with germination having no effect on the stability of the emulsions until day 2 of germination. Setia, Dai, Nickerson, Sopiwnyk, Malcolmson, and Ai [36] analyzed the impact of germination in pulse’s flours, reporting an improved EAI and ESI for the flours to a certain degree, due to the partial unfolding and dissociation of proteins, promoting surface activity. This effect could be attributed to an increased surface hydrophobicity of the compounds, which plays a key role in boosting emulsifying activities. Regarding the influence of the MW of the proteins, previous studies have reported that low-MW peptides can disperse oil globules more evenly by unfolding and rearranging the molecules at the oil–water interface, resulting in more surface hydrophobicity and a higher EAI [27]. Contrary to the results of this study, Ghumman, Kaur, and Singh [29] reported a decrease in the EAI with an increase in germination duration in lentil seeds, which could be attributed to an increase in β-sheets and a decrease in α-helix structures in the proteins. The inability of low-MW peptides to create a strong film around oil droplets at the oil–water boundary could lead to the reduced stability of the emulsion being produced [27].

#### 3.5.3. FE and FS

Foams are created by the adsorption of proteins in the air–water interface, resulting in a decrease in the surface tension. During the foaming process, proteins need to unfold and create a supportive film around air bubbles, helping to maintain the foam’s structure and prevent it from collapsing [2]. The FE of different MW fractions of ungerminated lentils ranged from 121.35 to 124.01% and these levels increased to 133.87–137.97% after 6 days of germination. In general, high-MW fractions of lentil protein isolates had higher FE ability compared to low-MW fractions, although the FE of these later fractions significantly increased (*p* < 0.05; F = 121049.217) during the process of germination. This fact could be explained by the higher capacity of high-MW peptides to interact at the air–water interface, in comparison to low-MW fractions [27]. Liu et al. [54] reported that germinating hemp seeds enhanced their foaming capacity. Specifically, the foaming capacity increased from levels of 69% recorded after 1 day of germination (comparable to non-germinated seeds) to levels of 111 and 107%, appreciated after 3 and 5 days of germination, respectively. This improvement could be attributed to increased protein solubility leading to an increased stabilization of air bubbles by the proteins.

In the case of FS values, the ability of foams to retain air in the form of bubbles for 30/60 min increased during germination. The results demonstrated that around 80–90% of the created foam was stable after 30 min and 70–80% of bubbles remained unchanged even after 60 min. Moreover, the fractions of low MW showed overall the lowest levels of FS compared to the high-MW counterparts, although the FS of these low-MW compounds also increased during the germination process. There are multiple studies reporting increased FS by limiting the hydrolysis of compounds [52,55,56]. Ghumman, Kaur, and Singh [29] reported that the foam capacity of lentil flour improved progressively due to germination (4 days), which could be attributed to the formation of β-sheets and the rearrangement of the α-helix in the proteins. To a large extent, the FS is affected by the surface rheological properties of interfacial films [52]. The observed increase in the FS of protein isolates of germinated lentils may be attributed to the partial unfolding of protein structures that occurred due to hydrolytic enzymes during sprouting. Enzymes activated by germination cause protein size reduction and the greater exposure of hidden hydrophobic groups to form stable films at the air–water interface [57]. Martínez, Sánchez, Patino, and Pilosof [52] reported that low degrees of hydrolysis (2%) in soy protein isolates enhanced surface activity and decreased the phase angle of the films which led to a delay in the collapse of the foam. However, further hydrolysis resulted in lower foam stability. Thus, it is likely that the limited hydrolysis of lentil proteins during the process of germination is probably responsible for the increased hydrophobicity of the compounds, allowing the formation of a stable surface film and enhancement of foam stability.

## 4. Conclusions

The current research into the ACE-I inhibitory activity, antioxidant activity, protein digestibility, cytotoxicity, and functional properties of distinct MW fractions of proteins isolated from lentil seeds over a 6-day period of germination yielded valuable insights into the functionality and future uses of these compounds. Overall, the protein digestibility, ACE-I inhibitory activity, and antioxidant activities (DPPH, ferrous chelating, hydroxyl radical, and FRAP activities) improved during the process of germination, with all of them being at their highest levels in the low-MW fractions (<3 kDa). Moreover, the cytotoxicity analysis using L929 cell lines in this study demonstrated that the germinated lentil proteins at low MWs exhibited a significant adverse effect on cell proliferation, whereas in the MW > 5 kDa fraction, no toxicity was observed. When exploring the functional properties of the lentil protein fractions, alterations in the WHC and FBC were appreciated during the process of germination that could be attributed to changes in the MW of the compounds during the process of germination. The emulsifying (EAI and ESI) and foaming abilities (FE and FS) were interesting as low-MW fractions had higher EAIs, while at the same time, they had considerably lower ESIs when compared to high-MW fractions; meanwhile, the FE and FS were both higher in high-MW fractions as compared to the low-MW ones. Nevertheless, further studies are necessary using the INFOGEST method to facilitate the comparison of the results. Further studies are also necessary to determine the specific mechanisms responsible for these changes and to analyze all aspects of their potential effects on human health and their interactions with other ingredients in other complex food formulations.

## Figures and Tables

**Table 1:** Summary of changes in proximate composition of lentil seeds during germination (0–6 days).

Germination Period (Days)	Moisture (%)	Protein (%)	Fat (%)	Carbohydrates (%)	Fiber (%)	Ash (%)
0	9.33 ± 0.00 ^e^	22.46 ± 0.01 ^e^	2.51 ± 0.01 ^a^	43.20 ± 0.01 ^a^	19.95 ± 0.01 ^e^	2.41 ± 0.02 ^c^
1	10.18 ± 0.01 ^d^	22.59 ± 0.02 ^d^	2.30 ± 0.01 ^a^	42.16 ± 0.01 ^b^	20.16 ± 0.00 ^d^	2.44 ± 0.04 ^bc^
2	10.67 ± 0.01 ^c^	22.91 ± 0.01 ^d^	2.26 ± 0.00 ^a^	41.07 ± 0.00 ^c^	20.33 ± 0.01 ^c^	2.57 ± 0.02 ^bc^
3	10.91 ± 0.00 ^bc^	23.34 ± 0.03 ^c^	1.94 ± 0.00 ^b^	40.38 ± 0.02 ^d^	20.68 ± 0.01 ^b^	2.69 ± 0.01 ^b^
4	11.90 ± 0.05 ^b^	24.37 ± 0.02 ^b^	1.18 ± 0.00 ^c^	38.19 ± 0.08 ^e^	21.31 ± 0.02 ^a^	3.00 ± 0.01 ^a^
5	12.25 ± 0.05 ^ab^	24.56 ± 0.04 ^ab^	1.12 ± 0.01 ^c^	37.44 ± 0.07 ^e^	21.44 ± 0.03 ^a^	3.44 ± 0.03 ^a^
6	12.73 ± 0.02 ^a^	24.71 ± 0.02 ^a^	1.08 ± 0.01 ^c^	36.66 ± 0.00 ^e^	21.52 ± 0.00 ^a^	3.52 ± 0.00 ^a^

Data are expressed as average ± standard deviation (n = 2). Different letters within each column indicate statistically significant differences (*p* < 0.05) for each parameter during 6 days of germination.

**Table 2:** Content of β-sheet of lentil protein during 6 days of germination.

Germination Period (Days)	Area Under Curve (AUC)	% β-Sheet
0	8.39	15.78
1	21.53	15.79
2	16.76	17.05
3	14.56	16.94
4	16.08	19.18
5	6.40	18.45
6	12.48	18.17

**Table 3:** Digestibility (%) of different MW fractions achieved from protein isolates generated from lentils during process of germination (days 0–6).

Germination Duration (Day)	MW (kDa)		
	MW > 5	3 < MW < 5	MW < 3
0	16.32 ± 0.01 ^Bg^	17.00 ± 0.01 ^Ag^	17.04 ± 0.00 ^Ag^
1	17.05 ± 0.01 ^Cf^	17.37 ± 0.00 ^Bf^	18.26 ± 0.03 ^Af^
2	18.75 ± 0.00 ^Ce^	19.22 ± 0.02 ^Be^	19.78 ± 0.01 ^Ae^
3	20.67 ± 0.03 ^Cd^	21.45 ± 0.02 ^Bd^	21.85 ± 0.04 ^Ad^
4	22.64 ± 0.02 ^Bc^	22.89 ± 0.01 ^Bc^	23.16 ± 0.01 ^Ac^
5	23.23 ± 0.01 ^Bb^	23.80 ± 0.00 ^Ab^	24.16 ± 0.02 ^Ab^
6	24.92 ± 0.02 ^Ca^	25.64 ± 0.02 ^Ba^	26.05 ± 0.03 ^Aa^

Data are expressed as average ± standard deviation (n = 2). Different uppercase letters indicate statistically significant differences (*p* < 0.05) in digestibility between fractions of different MW within same germination day. Lowercase letters indicate statistically significant (*p* < 0.05) differences in digestibility between different germination days at same MW.

**Table 4:** ACE-I inhibitory activity (IC_50_) of different MW fractions achieved from protein isolates from lentils during the process of germination (days 0–6).

Germination Period (Days)	MW (kDa)		
	MW > 5	3 < MW < 5	MW < 3
0	1.49 ± 0.02 ^Aa^	1.19 ± 0.01 ^Ba^	1.03 ± 0.00 ^Ba^
1	1.39 ± 0.00 ^Aa^	1.17 ± 0.02 ^Aa^	0.97 ± 0.01 ^Bab^
2	1.28 ± 0.01 ^Aab^	1.11 ± 0.01 ^Ba^	0.91 ± 0.00 ^Cab^
3	1.21 ± 0.01 ^Ab^	1.05 ± 0.01 ^Ba^	0.87 ± 0.01 ^Cb^
4	1.05 ± 0.01 ^Ac^	0.91 ± 0.00 ^Bb^	0.73 ± 0.02 ^Bc^
5	1.00 ± 0.01 ^Ac^	0.88 ± 0.01 ^Bbc^	0.71 ± 0.01 ^Cc^
6	0.95 ± 0.01 ^Ac^	0.83 ± 0.00 ^Ac^	0.69 ± 0.01 ^Bc^

Data are expressed as average ± standard deviation (n = 2). Different uppercase letters indicate statistically significant differences (*p* < 0.05) in ACE-I inhibitory activity between fractions of different MW within same germination day. Lowercase letters indicate statistically significant (*p* < 0.05) differences in ACE-I inhibitory activity between different germination days at same MW.

**Table 5:** Functional properties of different MW fractions generated from protein isolates from lentils during process of germination (days 0–6).

Germination Period (Days)	MW (kDa)	WHC (%)	FBC (%)	EAI (m^2^/g)	ESI (min)	FE (%)	FS (%)	
							30 min	60 min
0	MW > 5	93.24 ± 0.02 ^Fc^	97.67 ± 0.02 ^Gc^	101.32 ± 0.02 ^Gc^	25.32 ± 0.02 ^Aa^	124.01 ± 0.07 ^Ga^	67.10 ± 0.01 ^Ga^	54.60 ± 0.01 ^Ga^
0	3 < MW < 5	95.38 ± 0.01 ^Eb^	99.35 ± 0.01 ^Gb^	103.66 ± 0.03 ^Gb^	24.17 ± 0.01 ^Ab^	123.18 ± 0.01 ^Da^	64.34 ± 0.01 ^Gb^	52.11 ± 0.01 ^Gb^
0	MW < 3	97.89 ± 0.01 ^Ga^	106.31 ± 0.01 ^Ga^	105.12 ± 0.01 ^Fa^	22.18 ± 0.03 ^Ac^	121.35 ± 0.01 ^Gb^	63.17 ± 0.01 ^Gc^	51.45 ± 0.01 ^Gc^
1	MW > 5	94.68 ± 0.00 ^Ec^	98.65 ± 0.01 ^Fc^	102.32 ± 0.02 ^Fb^	25.02 ± 0.04 ^ABa^	126.84 ± 0.04 ^Fa^	69.47 ± 0.02 ^Fa^	55.12 ± 0.01 ^Fa^
1	3 < MW < 5	95.16 ± 0.01 ^Eb^	103.24 ± 0.01 ^Fb^	106.76 ± 0.35 ^Fa^	24.23 ± 0.01 ^Ab^	124.05 ± 0.01 ^Db^	66.22 ± 0.02 ^Fb^	53.28 ± 0.01 ^Fb^
1	MW < 3	98.37 ± 0.02 ^Fa^	108.96 ± 0.02 ^Fa^	109.55 ± 0.01 ^EFa^	22.08 ± 0.01 ^Ac^	122.73 ± 0.02 ^Fc^	65.81 ± 0.01 ^Fc^	52.25 ± 0.04 ^Fc^
2	MW > 5	95.09 ± 0.01 ^Dc^	103.24 ± 0.02 ^Ec^	105.44 ± 0.01 ^Ec^	24.98 ± 0.01 ^Ba^	128.13 ± 0.01 ^Ea^	71.18 ± 0.02 ^Ea^	59.49 ± 0.02 ^Ea^
2	3 < MW < 5	96.36 ± 0.03 ^Db^	108.46 ± 0.03 ^Eb^	108.66 ± 0.02 ^Eb^	23.44 ± 0.02 ^Bb^	125.18 ± 0.00 ^Db^	68.58 ± 0.04 ^Eb^	55.16 ± 0.03 ^Eb^
2	MW < 3	97.05 ± 0.01 ^Ea^	111.72 ± 0.03 ^Ea^	112.09 ± 0.01 ^Ea^	22.04 ± 0.02 ^Ac^	122.97 ± 0.02 ^Ec^	66.01 ± 0.01 ^Ec^	53.01 ± 0.03 ^Ec^
3	MW > 5	97.12 ± 0.02 ^Dc^	108.36 ± 0.01 ^Dc^	109.05 ± 0.01 ^Dc^	24.70 ± 0.01 ^Ca^	130.86 ± 0.03 ^Da^	75.83 ± 0.04 ^Da^	64.32 ± 0.01 ^Da^
3	3 < MW < 5	97.86 ± 0.01 ^Cb^	113.45 ± 0.01 ^Db^	113.25 ± 0.01 ^Db^	23.10 ± 0.01 ^Cb^	127.65 ± 0.01 ^Cb^	70.21 ± 0.01 ^Db^	58.50 ± 0.03 ^Db^
3	MW < 3	98.29 ± 0.01 ^Da^	117.24 ± 0.01 ^Da^	118.46 ± 0.02 ^Da^	21.74 ± 0.01 ^Bc^	124.11 ± 0.01 ^Dc^	69.45 ± 0.02 ^Dc^	54.13 ± 0.01 ^Dc^
4	MW > 5	97.51 ± 0.02 ^Cc^	109.33 ± 0.02 ^Cc^	114.80 ± 0.01 ^Cc^	24.16 ± 0.02 ^Da^	133.44 ± 0.05 ^Ca^	84.53 ± 0.02 ^Ca^	73.14 ± 0.01 ^Ca^
4	3 < MW < 5	98.47 ± 0.01 ^Bb^	116.65 ± 0.03 ^Cb^	118.07 ± 0.01 ^Cb^	22.91 ± 0.02 ^Cb^	130.12 ± 0.03 ^Bb^	76.92 ± 0.01 ^Cb^	68.37 ± 0.01 ^Cb^
4	MW < 3	99.815 ± 0.03 ^Ca^	121.33 ± 0.02 ^Ca^	124.96 ± 0.01 ^Ca^	20.53 ± 0.02 ^Cc^	128.03 ± 0.02 ^Cc^	72.14 ± 0.01 ^Cc^	60.19 ± 0.01 ^Cc^
5	MW > 5	98.22 ± 0.01 ^Bc^	116.29 ± 0.01 ^Bc^	119.35 ± 0.01 ^Bc^	23.95 ± 0.02 ^Da^	135.07 ± 0.01 ^Ba^	87.27 ± 0.03 ^Ba^	75.08 ± 0.01 ^Ba^
5	3 < MW < 5	101.35 ± 0.03 ^Ab^	120.78 ± 0.01 ^Bb^	124.55 ± 0.02 ^Bb^	22.45 ± 0.01 ^Db^	134.77 ± 0.02 ^Ab^	80.36 ± 0.02 ^Bb^	71.08 ± 0.01 ^Bb^
5	MW < 3	108.56 ± 0.01 ^Ba^	127.35 ± 0.02 ^Ba^	131.03 ± 0.04 ^Ba^	20.23 ± 0.02 ^CDc^	133.42 ± 0.02 ^Bc^	74.51 ± 0.01 ^Bc^	68.42 ± 0.01 ^Bc^
6	MW > 5	100.36 ± 0.35 ^Ac^	119.44 ± 0.01 ^Ac^	123.63 ± 0.02 ^Ac^	23.72 ± 0.01 ^Ea^	137.97 ± 0.05 ^Aa^	89.34 ± 0.03 ^Aa^	79.61 ± 0.02 ^Aa^
6	3 < MW < 5	109.55 ± 0.10 ^Ab^	125.28 ± 0.03 ^Ab^	129.13 ± 0.01 ^Ab^	22.29 ± 0.02 ^Db^	135.12 ± 0.02 ^Ab^	82.43 ± 0.05 ^Ab^	76.95 ± 0.02 ^Ab^
6	MW < 3	117.64 ± 0.10 ^Aa^	134.87 ± 0.03 ^Aa^	135.42 ± 0.01 ^Aa^	20.14 ± 0.02 ^Dc^	133.87 ± 0.02 ^Ac^	79.34 ± 0.05 ^Ac^	72.13 ± 0.02 ^Ac^

Data are expressed as average ± standard deviation (n = 2). Different uppercase letters indicate statistically significant (*p* < 0.05) differences between various germination days at same molecular weight. Lowercase letters indicate statistically significant differences (*p* < 0.05) in functional properties between fractions of different molecular weight within same germination day. Abbreviations in table are as follows: molecular weight (MW), water holding capacity (WHC), fat binding capacity (FBC), emulsion activity index (EAI), emulsion stability index (ESI), foam expansion (FE), and foam stability (FS).

## Data Availability

All data generated or analyzed during this study are included in this published article.
